# Timber and trails: Low‐intensity selective logging and elephant trails shape seedling dynamics in an Afrotropical forest

**DOI:** 10.1002/eap.70180

**Published:** 2026-02-01

**Authors:** Megan K. Sullivan, Luke Browne, Prince Armel Mouguiama Bissiemou, Raoul Niangadouma, Katharine Abernethy, Simon A. Queenborough, Liza S. Comita

**Affiliations:** ^1^ School of the Environment Yale University New Haven Connecticut USA; ^2^ High Meadows Environmental Institute Princeton University Princeton New Jersey USA; ^3^ Agence Nationale Des Parcs Nationaux Batterie IV Libreville Gabon; ^4^ Herbier National du Gabon Libreville Gabon; ^5^ African Forest Ecology Group, Faculty of Natural Sciences University of Stirling Stirling UK; ^6^ Institut de Recherche en Ecologie Tropicale Libreville Gabon; ^7^ Smithsonian Tropical Research Institute Ancón Republic of Panama

**Keywords:** African forest, elephant trails, lianas, OECM, seedling dynamics, selective logging, tropical forest

## Abstract

Very low‐intensity selective logging can be a compromise between strict conservation and income‐generating land use in tropical forests. Investigating how selective logging influences the understory environment and seedling dynamics as the forest regenerates offers insights into whether logging alters forest dynamics, influencing the composition and structure of future forests. We explored how very low‐intensity logging (<2 trees ha^−1^) influences understory factors and seedling dynamics across a logging chronosequence (unlogged forest vs. actively logged forest and forest logged 4 and 14 years prior). To do this, we assessed (1) how canopy openness, prevalence of vegetation damage, and elephant trails differ in logged forests at different recovery stages compared to unlogged forest; (2) how these understory factors influence seedling dynamics; (3) how seedling dynamics differ across the logging chronosequence; and (4) how logging impacts liana vs. tree seedlings across the chronosequence. We observed greater canopy openness and vegetation damage in logged forests up to 4 years after logging and higher elephant trail prevalence 14 years after logging compared to unlogged forests. Seedling survival was lower in plots with higher canopy openness, more vegetation damage, and on elephant trails, while growth and recruitment were not affected by these variables. Actively logged forests initially had lower seedling survival and recruitment, but higher growth rates compared to unlogged forests. However, 14 years after logging, seedling dynamics were mostly similar to unlogged forests. Liana seedlings had a slight growth advantage over tree seedlings in all logged forests compared to unlogged forests. Results from our study suggest that very low‐intensity selective logging causes temporary shifts in understory dynamics rather than long‐term shifts in forest recovery trajectories. These managed areas have potential as land that can contribute to OECM targets—functioning as mixed‐use corridors, connecting protected areas across a landscape and contributing to biodiversity and wildlife conservation—especially in countries with high forest cover and low deforestation.

## INTRODUCTION

Income‐generating land uses are often a threat to tropical forests, but they do not have to be (Burivalova et al., 2014; Ellis et al., 2019; Runting et al., 2019; Zwerts et al., 2024). Selective logging is one example of a limited economic use that has been championed in developing nations by organizations such as the Forest Stewardship Council (FSC) and the International Tropical Timber Organization (ITTO) (EDF, 2023; FSC, 2023; ITTO, 2024; Lescuyer et al., 2021; Romero et al., 2017). Selective logging is a compromise that can allow profitable land use while minimizing negative impacts on biodiversity (Chaudhary et al., 2016; Putz et al., 2012; Sullivan et al., 2022), carbon storage (Gourlet‐Fleury et al., 2013), and other ecosystem services (Malhi et al., 2022; Sullivan et al., 2024). The success of this compromise is dependent on the specific practices and intensity of logging, both of which vary widely across regions (Karsenty, 2016; Putz et al., 2019; Sist, 2000) and individual logging operators (Umunay et al., 2019; Zwerts et al., 2024). Thus, understanding the limits of sustainable logging is essential to the longevity of managed forests.

Most studies on the impacts of logging focus on how forest structure and biodiversity change after logging occurs. However, few assess how logging affects the mechanistic drivers and ecological processes that shape tree recruitment and establishment as the forest recovers from selective logging. Studying early life stages can elucidate which ecological processes set the stage for forest composition in the future (Pillay et al., 2018). Single‐census, space‐for‐time studies have limitations for understanding the ecological drivers behind pattern shifts because they only capture a snapshot in time (Addo‐Fordjour et al., 2022; Sullivan et al., 2022, 2024). Multiple metrics of plant performance can shift simultaneously in response to disturbance. For example, selective logging can initially negatively impact seedling survival due to damage from logging activities (Pillay et al., 2018). After logging activities cease, a flush of seedlings proliferate and grow because of the greater light availability and lower competition in the logged forest understory (de Carvalho et al., 2017). Eventually, seedling recruitment saturates the open understory and the forest canopy closes over (Duah‐Gyamfi et al., 2014). Seedling survival, growth, and recruitment happen simultaneously but each may respond to logging differently and on different timelines (Pillay et al., 2018). Thus, dynamic data on these performance metrics are needed to tease apart the ecological drivers of shifts in forest structure, composition, and function following selective logging.

Logging affects forest heterogeneity and dynamism, particularly when considering understory factors, such as (1) canopy openness, (2) vegetation damage, and (3) animal activity. Light availability is typically higher or more variable immediately after logging, due to greater canopy openness from logging gaps (Sullivan et al., 2022). As the canopy regenerates post‐logging, light levels in the recovering logged forest become more similar to light levels in unlogged forests (Asner et al., 2004; Sullivan et al., 2022; Yamada et al., 2014). This increase in light levels can temporarily improve growing conditions for seedlings. Vegetation damage, from falling debris and deadwood, is also more prevalent in logged forests than in unlogged forests (Malhi et al., 2021; Mills et al., 2023; Pfeifer et al., 2015; Roopsind et al., 2018). Animal activity also shifts in response to logging, notably among elephants. During active logging, elephants tend to avoid disturbed areas due to logging noise and human presence (Burivalova et al., 2021; Struhsaker et al., 1996). Elephants often return post‐logging, using skid trails to navigate regenerating forests that are flush with herbaceous food sources (Burivalova et al., 2021; Scalbert, Stiernon, et al., 2023; White et al., 1995). These shifts in movement can alter the density and distribution of elephant trails in the understory (Keany et al., 2024). Because elephant trails concentrate animal traffic, any change in the trails can alter the spatial patterns of trampling and herbivory (Struhsaker et al., 1996) and seed dispersal (Campos‐Arceiz & Blake, 2011) in the recovering forest understory. Thus, even when elephant populations remain stable in logged forests (Scalbert, Stiernon, et al., 2023; Yoh et al., 2024; Zwerts et al., 2024), changes in movement and trail patterns may influence seedling dynamics and regeneration patterns post‐logging (Piiroinen et al., 2017; Scalbert, Vermeulen, et al., 2023).

Altogether, canopy openness, vegetation damage and debris, and elephant trails can shape seedling dynamics in multiple ways. By creating gaps and skid trails in the forest and opening the forest canopy (Asner et al., 2004), selective logging creates brighter, hotter, drier microsites in the understory (Blonder et al., 2018). Seedlings in the tropical forest understory are often light limited (Augspurger, 1984; Chazdon, 1988), and these higher light levels can improve seedling survival and recruitment (Denslow et al., 1990). Simultaneously, increased vegetation damage and falling debris in logging‐disturbed areas can cause greater seedling mortality. Seedlings that escape the actual threat of falling debris might benefit from higher light and less competition from neighboring plants in these logging‐disturbed areas (Wright, 2002). By affecting animal movement, logging also alters elephant trail dynamics as the forest recovers. During active logging, animal avoidance might mean less physical damage to seedlings from trampling and herbivory and decreased seed dispersal, potentially enhancing seedling survival while reducing seedling recruitment in actively logged forests (Campos‐Arceiz & Blake, 2011; Lawes & Chapman, 2006; Paul et al., 2004; Rosin et al., 2017; Struhsaker et al., 1996). During forest recovery, we may see the opposite pattern: higher seedling mortality due to animal‐driven physical damage (Piiroinen et al., 2017; Rosin et al., 2017) and greater seedling regeneration due to increased animal seed dispersal (Beaune et al., 2013; Campos‐Arceiz & Blake, 2011; Chapman & Chapman, 1995).

Lianas, in particular, might benefit from selective logging (Schnitzer & Bongers, 2011) because they can take better advantage of the disturbed conditions in logged forests than trees for a few reasons. First, many lianas are light‐demanding (Lowe & Walker, 1977; Phillips & Gentry, 1994; Putz, 1984; Whitmore, 1989) and more drought tolerant than trees (Schnitzer et al., 2020, 2021; Schnitzer & Bongers, 2011). Thus, lianas may experience increased growth, survival, and recruitment rates following logging. Additionally, lianas are often more capable of surviving physical damage and can produce many clonal stems post‐disturbance (Pouteau et al., 2024; Putz, 1984; Rocha et al., 2020). Thus, lianas may benefit from the increased understory light heterogeneity and the higher rates of falling debris that occur after logging. Because lianas may have a competitive advantage over tree seedlings, they may proliferate post‐logging in ways that alter liana and tree interactions (Addo‐Fordjour & Afram, 2021). Previous work has found evidence of higher liana densities in the understory of low‐intensity selectively logged forests in Gabon (Sullivan et al., 2022). However, it is unclear which ecological mechanisms drive these patterns. Comparing liana and tree seedling dynamics following logging can thus help us better understand forest recovery trajectories.

In this study, we assessed how selective logging influences the understory environment and woody seedling dynamics across a logging chronosequence in northwestern Gabon to answer the following questions:

1. What are the immediate and longer term impacts of selective logging on environmental factors: canopy openness, prevalence of vegetation damage (i.e., due to logging activities or naturally occurring falling debris), and prevalence of elephant trails?
2. How do these environmental factors (canopy openness, vegetation damage, and elephant trails) influence seedling performance (survival, growth, and recruitment)?
3. What are the immediate and longer term impacts of selective logging on seedling performance?
4. Does logging have different immediate or longer term impacts on lianas vs. tree seedlings?

## METHODS

### Data

#### Study area and seedling censuses

We conducted this study in the Société Equatoriale d'Exploitation Forestière (SEEF), a 477,033 ha logging concession east of Monts de Cristal National Park (0°42′41″ N, 10°17′18″ E) in northwestern Gabon (Sullivan et al., 2022, 2024). The Monts de Cristal region is made up of undulating hills (300–1000 m) across approximately 20,000 km^2^ (Sunderland et al., 2004). Vegetation type in this area is Central African lowland evergreen rainforest, which is highly diverse and contains a mix of shade‐tolerant, light‐demanding, and commercially important species (Sullivan et al., 2024). *Aucoumea klaineana* is a common adult species in these forests—a wind‐dispersed, long‐lived pioneer species that is exploited for timber (Medjibe et al., 2011). The climate is equatorial with a bimodal climate regime: there is an intense dry season June–August and a less intense dry period December–February (Vande Weghe, 2008). Mean annual temperatures range from 24 to 26°C, and annual rainfall averages 2000–2500 mm in Monts de Cristal National Park, reaching up to 3000 mm in nearby areas (Sunderland et al., 2004; Vande Weghe, 2008).

**FIGURE 1:**
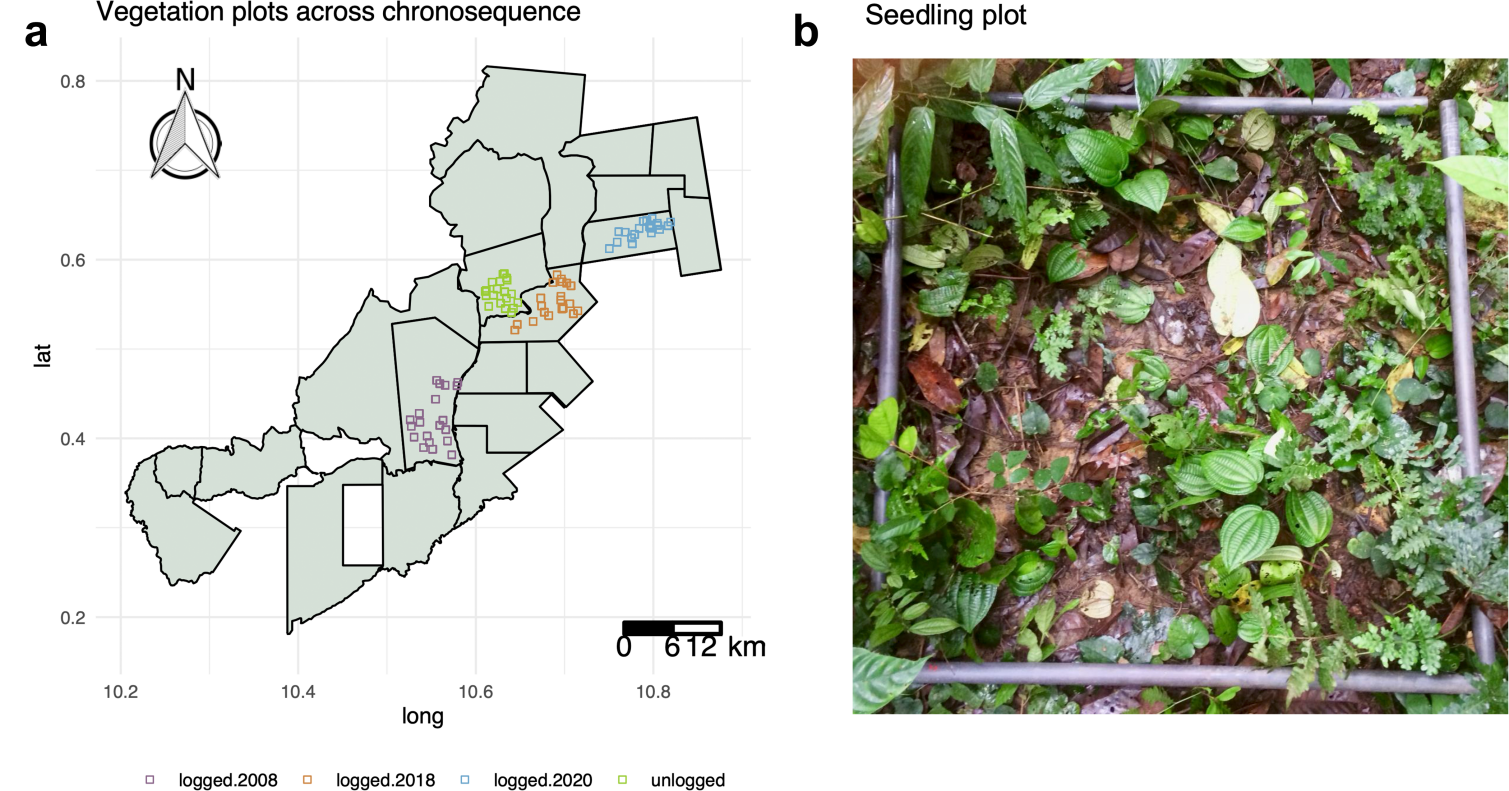
(a) Map showing the vegetation plot locations in actively logged (logged.2020), recently logged (logged.2018), older logged (logged.2008), and unlogged (control) treatments at our study site. Lat = latitude and Long = longitude. (b) Photo of a 1 × 1 meter seedling plot. Photo credit: Megan K. Sullivan.

SEEF began selective logging in 2000 at a low intensity (<2 trees ha^−1^; Medjibe et al., 2011; SEEF, 2019), which is typical for selective logging in the Congo Basin (Sist, 2000). To evaluate the impacts of logging and subsequent forest recovery, we established a logging chronosequence composed of eighty 20 × 20 m vegetation plots across four “blocks.” Each logging block was logged at a different time (in 2008, 2018, and 2020), and one block was an unlogged control area (Figure 1a, Table 1). Twenty plots were sampled per block. All plots were established and censused between September 2018 and October 2019 (Sullivan et al., 2022, 2024). Each plot contained nine 1 × 1 m seedling subplots (Figure 1b), for a total of 180 seedling subplots per block. During the first census, we tagged and measured the height of all woody seedlings ≤1 m tall in each seedling subplot. We re‐censused seedlings in February–September 2022. During that second census, we recorded the survival status (dead or alive) and height of each previously tagged seedling and tagged and measured new seedlings that had recruited into the seedling plots since the first census. We removed any observations of seedlings that were missing survival or height data before analyses. Tree seedlings were identified to species or morphospecies level, and liana seedlings were identified to the taxonomic level that was possible in the field. Any liana that lacked family level taxonomic information was labeled “liana” in the data (see Sullivan et al., 2022, 2024 for more details). –See full dataset for further information (Sullivan et al. 2025).

**TABLE 1:** The timing of logging, seedling censuses, and time since logging during each of the censuses.

Logging block	First census	Time since logging during first census	Second census	Time since logging during second census
Logged.2008	2018–2019	~10 years	2022	~14 years
Logged.2018	2018–2019	6 months–1 year	2022	~4 years
Logged.2020	2018–2019	Unlogged in first census	2022	~2 years (actively logged in between first and second census)
Unlogged	2018–2019	Unlogged	2022	Unlogged

#### Environmental factors and elephant trails

To quantify understory light availability, we measured canopy openness using a Spherical Crown Densiometer (Concave Model C, Forestry Suppliers, Jackson, MS, USA) (Lemmon, 1956) at a height of 1 m in the center of each 1 × 1 m seedling subplot. In each 1 × 1 m seedling subplot, we also recorded the presence/absence of vegetation damage from falling woody debris or skid trails and the presence/absence of elephant trails. All environmental factors and elephant trails were assessed during the 2022 census period.

### Statistical analyses

#### Selective logging impacts understory environmental factors

To examine differences in the understory environment among logging treatments, we used a mixed‐effects modeling approach to test for differences in each of the three environmental factors (mean canopy openness, presence/absence of vegetation damage, and presence/absence of elephant trails in each seedling plot) as a function of logging treatment (actively logged [logged.2020], recently logged [logged.2018], and older logged [logged.2008]) compared to the unlogged forest. In the models of presence/absence of elephant trails and vegetation damage, we used generalized linear mixed‐effects models (GLMMs) with binomial errors. In the models of mean canopy openness, we initially used a Poisson distribution, but due to overdispersion, we used a negative binomial distribution. In all three models, we included 20 × 20 m vegetation plot as a random effect to account for spatial autocorrelation and fit all models using the bobyqa optimizer in the lme4 package (Bates et al., 2015). Residuals were assessed for overdispersion using the DHARMa package (Hartig, 2024).

#### Seedling performance models

To test for effects of environmental variables and logging on the performance of woody seedlings, we modeled seedling survival, growth, and recruitment also using mixed‐effects models. Survival was a binomial response variable (1 = alive, 0 = dead), modeled using a GLMM with binomial errors. We calculated recruitment by adding up the total number of new seedling recruits per 1 × 1 m seedling plot across all species, modeled using a GLMM with negative binomial errors. For growth, we calculated relative growth rates of seedlings that survived to the second census as:

$$\mathrm{RGR}=\left(\log\left(\text{height}2\right)-\log\left(\text{height}1\right)\right)/\left(\text{time}\right)$$

where height2 = height at time two, height1 = height at time one, and time = the number of years between the first census and second census. Because relative growth rates exhibited a leptokurtic distribution and included both positive and negative values (due to stem breakage), we used a modulus transformation (John & Draper, 1980), as suggested in Condit et al. (2017), with a lambda value of 0.6 to transform growth rates and reduce skewness in the growth rate data. Modulus‐transformed growth was modeled using a linear mixed‐effect model (i.e., normal errors). In growth and survival models, we included the log‐transformed height of the individual in the last census as a fixed effect to account for size‐based differences in survival and growth probabilities. We included 20 × 20 m vegetation plot and 1 × 1 m seedling subplot as a random effect to account for spatial autocorrelation, and species as a random effect to account for species‐level differences in seedling performance in survival and growth models. In recruitment models, we included 20 × 20 m vegetation plot as a random effect to account for spatial autocorrelation. Recruitment was aggregated across all species in the recruitment response, because these forests are extremely diverse and many species occurred too infrequently to evaluate species‐specific recruitment with adequate statistical power. Thus, we could not include species as a random effect in our recruitment models. We fit all models using the bobyqa optimizer. Residuals were assessed for overdispersion using the DHARMa package.

For survival models, results are expressed in risk ratios. Risk ratios compare the likelihood of an event (in this case, survival) among one group with another group. A risk ratio greater than 1.0 indicates an increased likelihood of survival. A risk ratio less than 1.0 indicates a decreased likelihood of survival, that is, an increased risk of mortality. For recruitment models, results are expressed in incidence rate ratios. An incidence risk ratio indicates how much more (or less) commonly an event occurred in a focal group. Ratios higher than 1 mean that recruitment occurred more frequently in response to the explanatory variable, and ratios lower than one mean recruitment occurred less commonly. For example, a ratio of 3 means that recruitment occurred at 3 times the rate in the focal group (e.g., if the seedling is in a plot where elephant trail = 1) than in the non‐focal group (e.g., if the seedling is in a plot where elephant trail = 0).

#### Understory environmental factors impact seedling performance

We first tested how environmental factors (canopy openness, vegetation damage, and elephant trails) impacted seedling performance (survival, growth, and recruitment), using a full model with all environmental predictors. Because environmental factors were weakly correlated (Figure S1, Appendix S1: Table S2), we assessed whether multicollinearity among our environmental factors might impact our results. To do this, we calculated variance inflation factors (VIF) and tolerance values using the performance package (Lüdecke et al., 2021). All VIF values were <1.1, and all tolerance values were >0.9, indicating that multicollinearity was not strong enough to bias our model estimates (Appendix S1: Table S4).

#### Logging impacts seedling performance

Next, we tested how selective logging impacts seedling performance immediately after logging and over time by comparing each logging treatment to the unlogged forest treatment. We modeled our seedling performance metrics (survival, growth, and recruitment) separately as a function of logging treatment (actively logged [logged.2020], recently logged [logged.2018], and older logged [logged.2008]) compared to the unlogged forest. To test whether selective logging impacts lianas vs. trees differently, we modeled each seedling performance metric separately as a function of an interaction between the growth form of the seedling (liana = 1 vs. tree = 0) and logging treatment.

## RESULTS

### Selective logging impacts understory environmental factors

**FIGURE 2:**
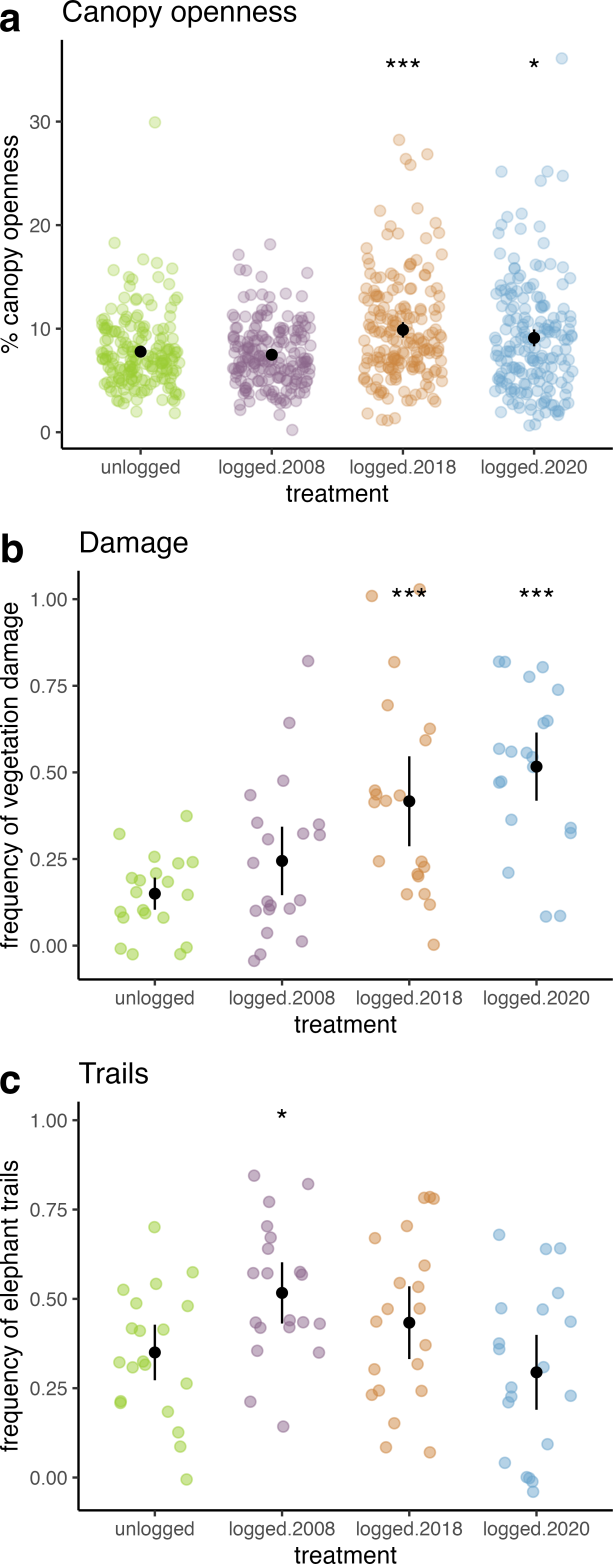
Differences in (a) canopy openness, (b) vegetation damage, and (c) elephant trails in subplots per plot between each logging treatments and the unlogged treatment. Colored points refer the average (a) percent canopy openness, (b) frequency of logging and vegetation damage, and (c) frequency of elephant trails in per vegetation plot. Black points represent the mean value, and black lines represent two standard errors around the mean, calculated from the data. ***, **, and * denote significant differences at the *p* < 0.001, 0.01, and 0.05 level, respectively.

We found that mean canopy openness was higher (IRR = 1.16, 95% CI: 1.00–1.33) in plots that were actively logged during the census interval [logged.2020] and higher (IRR = 1.26, 95% CI: 1.10–1.46) in the recently logged forest [logged.2018] compared to unlogged forest, but similar in the older logged [logged.2008] (IRR = 0.97, 95% CI: 0.84–1.11) compared to unlogged forest (Figure 2a, Appendix S1: Table S1a). Additionally, canopy openness was higher (IRR = 1.13, 95% CI: 1.05–1.22) in plots with vegetation damage and similar (1.06, 95% CI: 0.99–1.14) in plots with elephant trails compared to plots without damage or trails, respectively (Figure S1, Appendix S1: Table S2). Vegetation damage was 7.26 (95% CI: 3.47–15.21) times more likely to be observed in the area that was actively logged during the census interval [logged.2020], 4.59 (95% CI: 2.18–9.65) times more likely to be observed in the recently logged forest [logged.2018], and similar (1.84, 95% CI: 0.86–3.93) in the older logged forest [logged.2008] compared to the unlogged forest (Figure 2b, Appendix S1: Table S1b). Elephant trails were 2.09 (95% CI: 1.18–3.72) times more likely to be observed in the older logged forest [logged.2008] compared to unlogged forest, but similar in the actively logged [logged.2020] (0.69, 95% CI: 0.39–1.23) and recently logged [logged.2018] (1.45, 95% CI: 0.83, 2.55) forests compared to unlogged forest (Figure 2c; Appendix S1: Table S1c).

### Understory environmental factors impact seedling performance

**FIGURE 3:**
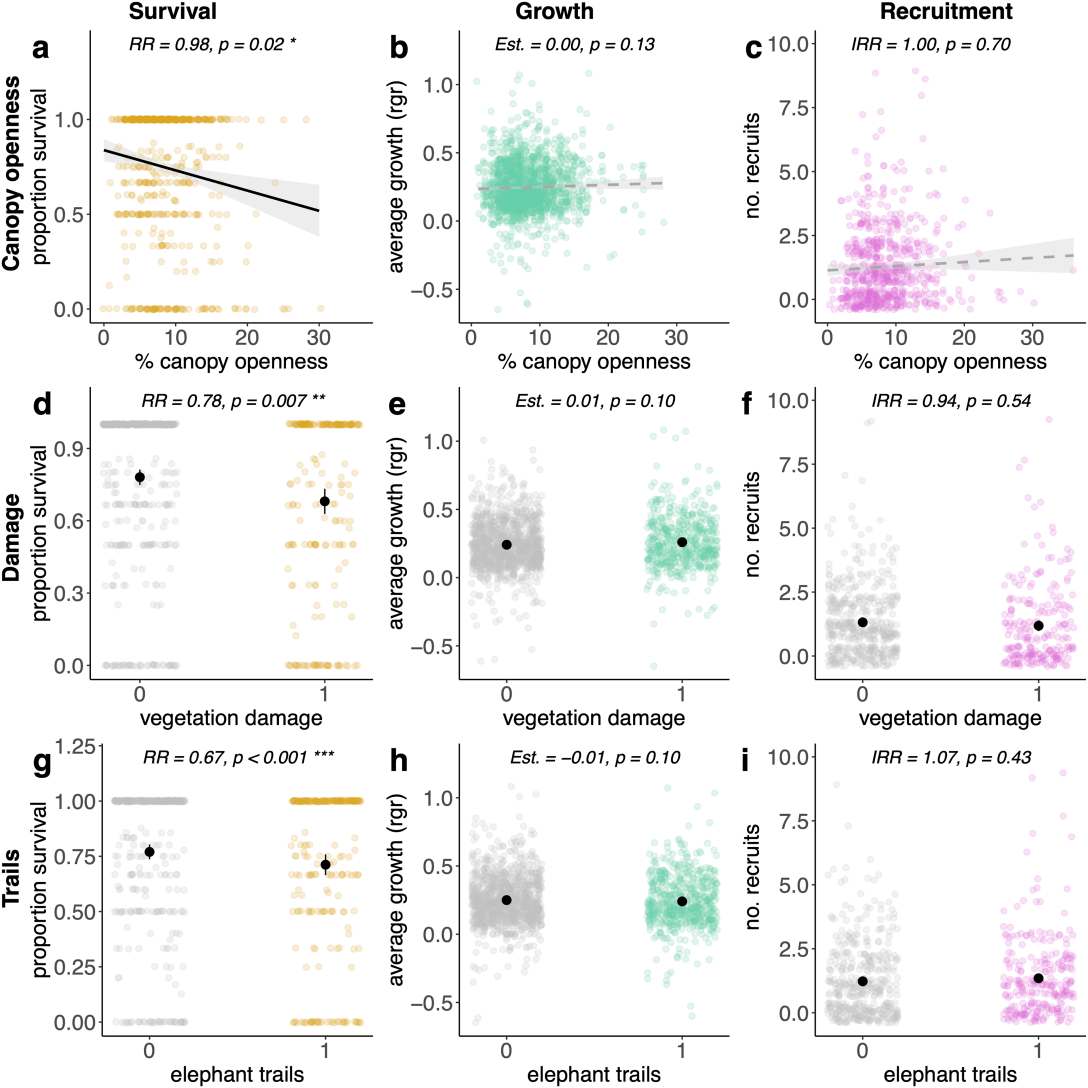
Coefficient plots (a‐i) show how canopy openness, vegetation damage, and elephant trails impacted seedling performance for survival, growth, and recruitment. Colored points represent values per 1 × 1 m seedling plot. Black points represent the mean value, and black lines represent two standard errors around the mean, calculated from the data. Summary statistics at the top of each panel are from full generalized linear models (i.e., all three predictors were included in each of the survival, growth, and recruitment models). ***, **, and * denote significant differences at the *p* < 0.001, 0.01, or 0.05 level, respectively. Rgr = relative growth rate.

Seedlings in plots with higher canopy openness were significantly less likely to survive (RR = 0.98, 95% CI: 0.96–1.00, Figure 3a, Appendix S1: Table S3a). Growth (Est = 0.00, 95% CI: −0.00–0.00) and recruitment (IRR = 1.00, 95% CI: 0.98–1.02) were similar across plots with different levels of canopy openness (Figure 3b,c, Appendix S1: Table S3b,c). Seedlings in plots with vegetation damage were significantly less likely to survive (RR = 0.78, 95% CI: 0.65–0.93) than seedlings in undamaged plots (Figure 3d, Appendix S1: Table S3a). Growth and recruitment were similar in damaged and undamaged plots (Growth: Est. = 0.01, 95% CI: −0.00–0.03, Figure 3e, Appendix S1: Table S3b) (Recruitment: IRR = 0.94, 95% CI: 0.77–1.14, Figure 3f, Appendix S1: Table S3c). Seedlings in plots with elephant trails were significantly less likely to survive (RR = 0.67, 95% CI: 0.57–0.80) than in plots without elephant trails (Figure 3g, Appendix S1: Table S3a). Growth and recruitment were similar in plots with and without elephant trails (Growth: Est. = − 0.01, 95% CI: −0.03–0.00, Figure 3h, Appendix S1: Table S3b) (Recruitment: IRR = 1.07, 95% CI: 0.90–1.28) (Figure 3i, Appendix S1: Table S3c).

### Logging impacts seedling performance

**FIGURE 4:**
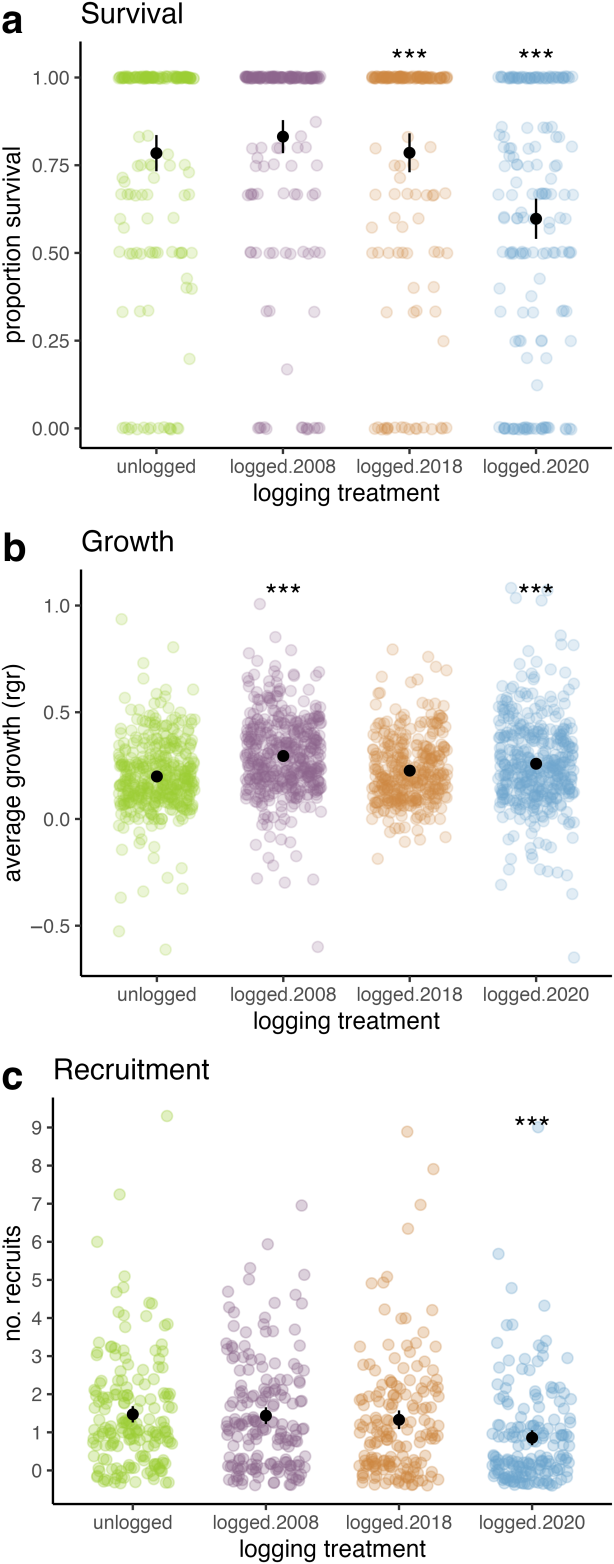
Differences in (a) survival, (b) growth, and (c) recruitment between each logging treatments and the unlogged treatment. In (a), colored points refer to the proportion of seedling survival per seedling plot. In (b), colored points refer to the relative growth rate of seedlings. In (c), colored points refer to the number of seedling recruits per seedling plot. Black points represent the mean value, and black lines represent two standard errors around the mean, calculated from the data. *** and * denote significant differences at the *p* < 0.001 and 0.05 level, respectively.

Overall, logging had negative impacts on seedling survival and recruitment, and positive impacts on seedling growth. We observed these logging impacts mostly in the active and recently logged forests (logged.2020 and logged.2018). Specifically, seedlings in plots that were actively logged during the recensus interval (logged.2020) were 0.47 (95% CI: 0.32–0.68) times less likely to survive, and seedlings in plots that were recently logged (logged.2018) were 0.65 (95% CI: 0.44–0.95) times less likely to survive as seedlings in unlogged plots (Figure 4a, Appendix S1: Table S5a). Growth was higher in actively logged plots (logged.2020: 0.06, 95% CI: 0.03–0.09) and older logged plots (logged.2008: 0.09, 95% CI: 0.07–0.12) compared to unlogged plots (Figure 4b, Appendix S1: Table S5b). Recruitment in actively logged plots (logged.2020) was 0.54 (95% CI: 0.38–0.75) times lower than in unlogged plots (Figure 4c, Appendix S1: Table S5c).

**FIGURE 5:**
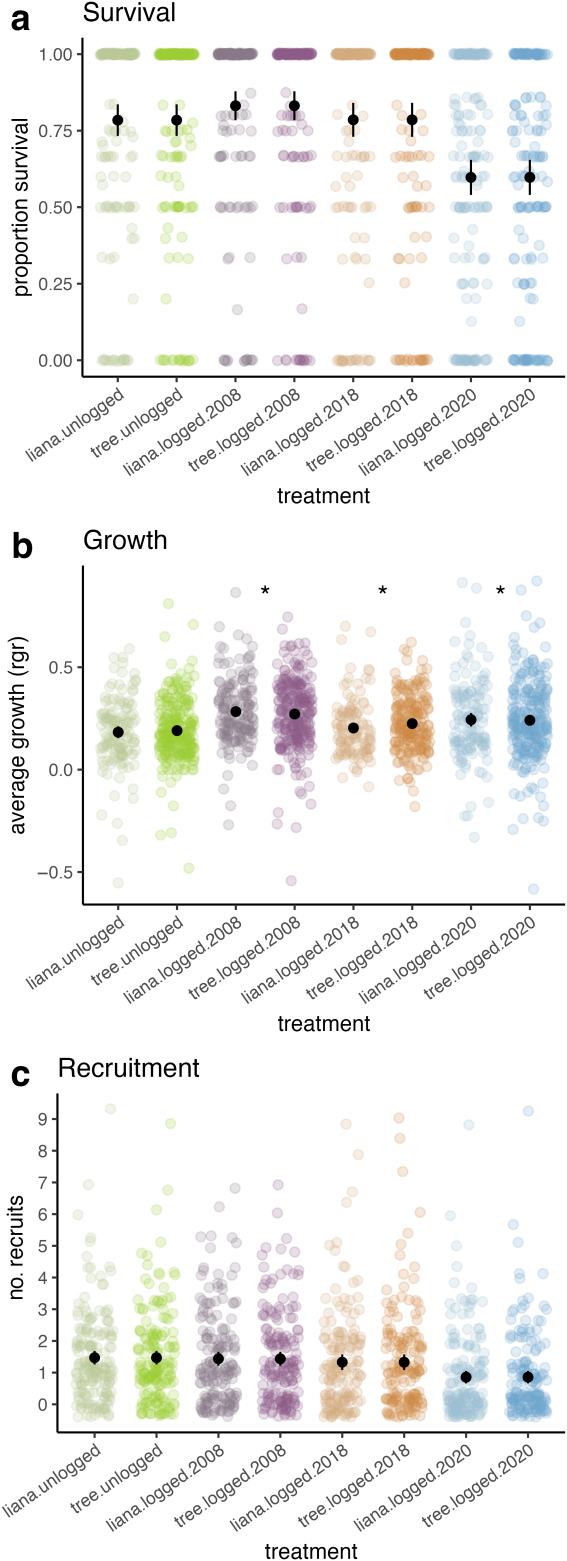
Differences in liana versus tree (a) survival, (b) growth, and (c) recruitment between each logging treatments and the unlogged treatment. In (a), colored points refer to the proportion of seedling survival per seedling plot. In (b), colored points refer to the relative growth rate of seedlings. In (c), colored points refer to the number of seedling recruits per seedling plot. Black points represent the mean value, and black lines represent two standard errors around the mean, calculated from the data. * denotes significant differences at the *p* < 0.05 level.

Lianas had similar survival rates as trees overall, and similar survival and recruitment responses to logged treatments compared to unlogged treatments (Figure 5a,c, Appendix S1: Table S6a,c). Overall, liana recruitment was 0.45 (95% CI: 0.33–1.62) times lower than trees across all plots (Appendix S1: Table S6c). Liana seedling growth was similar to tree growth overall, but liana seedlings grew more than tree seedlings in all of the logged treatments (logged.2020: 0.04, 95% CI: 0.00–0.07; logged.2018: 0.04, 95% CI: 0.00–0.08; logged.2008: 0.04, 95% CI: 0.00–0.07) compared to the unlogged treatment (Figure 5b, Appendix S1: Table S6b).

## DISCUSSION

We evaluated the effects of very low‐intensity selective logging on understory dynamics across a chronosequence in a Central African tropical forest. Our results indicate that low‐intensity selective logging can cause some shifts in understory environmental factors—canopy openness, vegetation damage, and elephant trails. The strongest impacts occurred up to 4 years after logging for vegetation damage and canopy openness and 14 years after logging for elephant trails. Moreover, we found that vegetation damage and elephant trails appear to have the strongest impact on seedling performance. Finally, our findings indicate that selective logging appears to only temporarily impact a limited cohort of regenerating and establishing seedlings. Specifically, seedling growth, mortality, and recruitment rates in logged forests were similar to rates observed in unlogged forests 4–14 years after logging activities ceased. Our findings add to discussions about the role of natural disturbance (Keany et al., 2024; Pouteau et al., 2024; Scalbert, Vermeulen, et al., 2023) and human management (Levis et al., 2017; Morin‐Rivat et al., 2017; Pouteau et al., 2024) in shaping tropical forests. Additionally, our results highlight the potential for these managed forests to contribute to biodiversity conservation goals and to maintain key ecosystem services.

### Environmental differences in logged versus unlogged forests

We observed elevated mean canopy openness and vegetation damage up to 4 years post‐logging. These differences dissipate and appear to return to similar levels as unlogged forests sometime between 4 and 14 years after logging occurred (Figure 2a,b). Our results indicate that while the initial damage from logging activities temporarily changes abiotic factors in the forest understory, these impacts diminish over time as the forest recovers from logging.

Our finding of elevated canopy openness is similar to other studies on gap dynamics post‐logging, which found that canopy gaps persisted at least 5 years after logging occurred (Asner et al., 2004). Prior studies also indicate that damaged trees, logging debris, and deadwood can continue to fall in forests anywhere from 4 to 10 years after logging occurs (Malhi et al., 2021; Mills et al., 2023; Pfeifer et al., 2015; Roopsind et al., 2018). Our findings are on the lower end of this spectrum, perhaps because the low logging intensity at our study sites causes less damage and recovery is faster. Overall, our results align with other studies that have found changes in the understory environment in low‐intensity selectively logged forests to be sparse and heterogeneous (Putz et al., 2019). These impacts of logging on the understory environmental factors we measured were less persistent than logging's impact on biodiversity, carbon storage metrics, or composition at this study site (Sullivan et al., 2022, 2024), as well as in other areas (Addo‐Fordjour et al., 2022; Osazuwa‐Peters et al., 2015).

Our results suggest that an increase in the prevalence of elephant trails in recovering logged forests is a lagged effect, which occurs anywhere from 4 to 14 years after logging ceases. Our results align with other studies that have reported increased elephant presence in recovering logged forests (Clark et al., 2009; Merz, 1981; Omeja et al., 2016; Poulsen et al., 2011; Struhsaker et al., 1996), and have found that elephants and other large mammals use old logging roads and skid trails to move through the forest (Keany et al., 2024; Kleinschroth & Healey, 2017; Scalbert, Stiernon, et al., 2023). To better understand how and why the prevalence of elephant trails in the understory varies over recovery time in logged forests, future studies could investigate what direct logging impacts (e.g., logging noise, human presence—Burivalova et al., 2021), and indirect logging impacts (e.g., deadwood and debris, herbaceous regrowth—White et al., 1995; Scalbert, Stiernon, et al., 2023; skid trails—Keany et al., 2024) are driving differences in forest use by elephants. Research about selective logging's impact on elephants has been inconclusive, with neutral (Scalbert et al., 2025; van Vliet & Nasi, 2008), mixed (Scalbert, Vermeulen, et al., 2023; Stokes et al., 2010), and negative results (Maisels et al., 2013) reported. Some of the differences in the results of these studies might be explained by unaccounted‐for variation between study sites, such as the synergistic impacts of both logging and hunting (Blake et al., 2007) and impacts from other human settlements within or nearby logging concessions (Lhoest et al., 2020).

### Environmental factors influence seedling performance

#### Canopy openness and vegetation damage

We found no effect of understory canopy openness on seedling growth or recruitment (Figure 3b,c). Instead, we found lower seedling survival with increasing canopy openness (Figure 3a). Additionally, seedling survival was lower in plots with vegetation damage compared to plots without (Figure 3d). Together, these results suggest that these two abiotic factors that change in logged forests—light and vegetation damage—play an important role in shaping seedling dynamics in the forest understory. Spatial associations between vegetation damage and light mean that we need to consider the effects of these impacts simultaneously (Figure S1, Denslow et al., 1990). Logging gaps are areas that have both higher light (Asner et al., 2004) and higher prevalence of vegetation damage (Malhi et al., 2021; Mills et al., 2023; Pfeifer et al., 2015; Roopsind et al., 2018), thus seedling mortality in logging gaps may be associated with both vegetation damage and higher light due to the spatial overlap of these two environmental factors (Figure S1). This association would explain why we found that seedlings in plots with higher canopy openness had higher mortality (Figure 3a). Because light and vegetation damage both impact seedling performance, performance metrics will likely be impacted for the amount of time that these factors are altered post‐logging—which was 4 years at our site, but could be longer if logging intensity is greater or management practices are different.

#### Elephant trails

Megafauna, such as elephants, act as ecosystem engineers in tropical forests, through behaviors such as trampling, browsing, and seed dispersal. However, understanding how the contrasting risks of seedling and tree mortality and benefits of seed dispersal shape community assembly processes is still not well understood (Scalbert, Vermeulen, et al., 2023). We found lower seedling survival in plots with elephant trails compared to plots without elephant trails (Figure 3g), but no evidence that seedling recruitment was enhanced in plots with elephant trails compared to plots without elephant trails (Figure 3g). These results provide important information about elephants' role in shaping forest dynamics at early life stages and elucidate imbalances between dispersal benefits and trampling risks.

Animal seed dispersal, broadly, is critically important for plant recruitment in tropical forests (Beckman & Rogers, 2013) and can have cascading impacts on biodiversity and carbon storage (Burak et al., 2024; Culot et al., 2017; Duvall et al., 2024). Simulation models based on forest elephant feeding preferences suggest that forests where elephants are present have 6%–9% higher aboveground carbon stocks compared to forests where elephants are absent (Berzaghi et al., 2023). These models simulated the benefits of elephant‐dispersed seeds and their eventual effects on forest carbon stocks but did not account for the increased risk of trampling that elephant‐dispersed seeds may experience by virtue of being dispersed into heavily trafficked areas, such as on elephant trails (Benitez & Queenborough, 2021) and around fruiting trees (Rosin et al., 2020). Our findings align with other studies that have found that, in heavily trafficked areas, damage from trampling, digging, rooting, and herbivory means that seedlings are more likely to be trampled or killed (Piiroinen et al., 2017; Rosin et al., 2017; Short, 1981).

Currently, there is a strong focus in the tropical forest literature on animal seed dispersal and seed arrival at different sites. However, the impacts of animal‐driven physical damage on regeneration success and seedling establishment have received comparatively much less attention (Piiroinen et al., 2017; Rosin et al., 2017; Scalbert, Vermeulen, et al., 2023). Future studies could use experimental approaches to directly quantify the impacts of different plant–animal interactions, such as plant damage and mortality (Beck et al., 2013; Terborgh & Wright, 1994) and seed fate post‐dispersal (Forget & Wenny, 2005; Rosin et al., 2017). Comparing the magnitude of impact that these processes have in logged and unlogged forests would allow us to better understand if, when, and how selective logging changes plant–animal interactions in managed forests.

### Effects of selective logging on seedling performance

We expected to find both immediate and longer term impacts of selective logging on seedling dynamics because of the environmental changes occurring in logged forests (Figure 2) that affect seedling performance (Figure 3). Our results suggest that the strongest impacts on seedling dynamics in logged forests are seen up to 4 years post‐logging (Figure 4) and appear to be driven by logging damage, as well as increased canopy openness and damage from falling debris in recently logged forests. These changes impact seedling survival and recruitment patterns, which can leave a temporary signature of logging in the early life stages of the recovering logged forest. However, seedling dynamics in older logged forests appear to be more similar to seedling dynamics in unlogged forests (Figure 4), indicating that the ecological drivers of seedling dynamics are not being permanently altered in the selectively logged forests we studied, similar to findings from previous work at this site (Sullivan et al., 2022, 2024).

Linking changes in seedling dynamics over time to environmental shifts in recovering logged forests can help us better understand how long it takes forests to recover (Fayolle et al., 2015; Gourlet‐Fleury et al., 2013; Osazuwa‐Peters et al., 2015). This information can allow us to predict the signatures of logging in forest vegetation, how persistent those signatures are, and can help us identify management strategies that could help promote forest recovery after logging.

Additionally, emerging research highlights how simultaneous disturbances—such as logging, elephants, and fire—may contribute to the maintenance of alternative stable states within Central African forest ecosystems. Examples include Marantaceae‐dominated understory patches (Picard, 2024; Pouteau et al., 2024) and forest edges (Cardoso et al., 2020), natural forest clearings called baïs (Hockridge et al., 2024; Turkalo et al., 2013), and herb‐dominated logging gaps (Lawes & Chapman, 2006; Paul et al., 2004). Incorporating multiple, interacting disturbances into our assessments of the impact of selective logging disturbance may be critically important to inform the management of logged tropical forests with intact megafauna. Framing the impacts of very low‐intensity selective logging in the context of both other natural disturbance regimes (Cardoso et al., 2020; Keany et al., 2024; Scalbert, Vermeulen, et al., 2023) and historical human disturbance regimes (Cardoso et al., 2020; Levis et al., 2017; Morin‐Rivat et al., 2017; Pouteau et al., 2024) may help clarify how the impacts of logging compare to disturbance mechanisms that have shaped Central and West African forests for millennia.

#### Logging impacts on lianas versus trees

Overall, our results indicate that the open, brighter, and more damage‐prone environment of the recently logged forests confers a small competitive growth advantage to liana compared to tree seedlings (Figure 5b). However, logging does not seem to be generating any seedling survival or recruitment benefits for lianas relative to trees, because survival and recruitment were similar in logged and unlogged forests for both groups (Figure 5a,c).

In a previous study at this site, we found higher relative abundances of lianas in the sapling stage of older logged forests compared to unlogged forest (Sullivan et al., 2022). The growth advantage that liana seedlings have in forests immediately after logging could help explain the higher relative abundances of lianas in later life stages as the logged forest recovered. Alternatively, lianas could also have a competitive advantage over trees at larger life stages, that is, via vegetative or clonal recruitment of sapling‐sized lianas in canopy gaps (Schnitzer et al., 2021), similar to the vegetative proliferation of large tree‐like herbs in the Marantaceae forests of the region (Brugière et al., 2000; Gillet, 2013; Pouteau et al., 2024; White et al., 1995). Inquiries into whether vegetative or clonal liana recruitment is occurring at later life stages would help better understand the drivers of increasing lianification of logged forests (Rueda‐Trujillo et al., 2024).

## CONCLUSIONS

Our study provides evidence that very low‐intensity selectively logged forests can be a compromise that balances the economic development and environmental protection of tropical forests (Ellis et al., 2019; Putz et al., 2012; Runting et al., 2019). Our results indicate that low‐intensity selective logging causes temporary shifts in some abiotic factors—canopy openness and vegetation damage—most strongly up to 4 years after logging occurs. These understory environmental factors impact seedling dynamics as logged forests recover, which can explain why we see the strongest differences in seedling performance in logged forests compared to unlogged forests up to 4 years post‐logging. Elephant trails were also more prevalent in 14‐year‐old logged forests, indicating that high conservation priority wildlife species increase forest use as the forest recovers. Both elephant trails and logging play key roles in shaping early‐stage forest processes, highlighting the need for more studies examining how overlapping disturbances interact to influence community assembly processes. Although the most important consideration for the strict protection of forests should be to maintain unlogged forests within protected areas with active monitoring and enforcement, our study presents convincing evidence that very low‐intensity logging limits the potential negative environmental impacts and does not appear to cause dramatic, long‐term shifts in the ecological processes of these managed forests. Thus, if managed carefully, these forests have the potential to extend the land area outside of formally protected areas that can meaningfully contribute to conservation goals.

Recent research has highlighted the potential for very low‐intensity logged forests to be designated as “Other Effective Area‐Based Conservation Measures” (OECMs)—mixed‐use areas that support conservation objectives while allowing sustainable use (CBD, 2018; Sullivan et al., 2024; Zwerts et al., 2024). Our findings support this view by showing that low‐intensity selective logging has limited and short‐lived impacts on understory conditions and seedling performance, suggesting that such forests can maintain ecological processes vital to biodiversity conservation. In High Forest Cover Low Deforestation countries, where development pressures are increasing, recognizing and managing these low‐intensity logged forests as OECMs could help protect wildlife, sustain carbon stocks, and connect protected areas via forest corridors (EDF, 2023)—provided that anti‐poaching enforcement and local socioeconomic contexts are effectively addressed (Blake & Maisels, 2023).

## AUTHOR CONTRIBUTIONS

Megan K. Sullivan, Simon A. Queenborough, and Liza S. Comita designed the study, and Megan K. Sullivan, Luke Browne, Simon A. Queenborough, and Liza S. Comita conducted data analysis. Megan K. Sullivan, Prince Armel Mouguiama Bissiemou, and Raoul Niangadouma collected, processed, and organized data. Megan K. Sullivan wrote the manuscript with input from Katharine Abernethy, Simon A. Queenborough, and Liza S. Comita.

## CONFLICT OF INTEREST STATEMENT

The authors declare no conflicts of interest.

## Supporting information


Appendix S1:

## References

[eap70180-bib-0001] Addo‐Fordjour, P. , and I. S. Afram . 2021. “Clearcutting and Selective Logging Have Inconsistent Effects on Liana Diversity and Abundance but Not on Liana–Tree Interaction Networks.” Biotropica 53: 442–452. 10.1111/btp.12888.

[eap70180-bib-0002] Addo‐Fordjour, P. , I. S. Afram , and J. Oppong . 2022. “Selective and Clear‐Cut Logging Have Varied Imprints on Tree Community Structure in a Moist Semi‐Deciduous Forest in Ghana.” Heliyon 8, 11. 10.1016/j.heliyon.2022.e11393. PMC9649955 36387494

[eap70180-bib-0003] Asner, G. P. , M. Keller , J. Pereira , Rodrigo , J. C. Zweede , and J. N. M. Silva . 2004. “Canopy Damage and Recovery after Selective Logging in Amazonia: Field and Satellite Studies.” Ecological Applications 14: 280–298. 10.1890/01-6019.

[eap70180-bib-0004] Augspurger, C. K. 1984. “Seedling Survival of Tropical Tree Species: Interactions of Dispersal Distance, Light‐Gaps, and Pathogens.” Ecology 65: 1705–1712. 10.2307/1937766.

[eap70180-bib-0006] Bates, D. , M. Mächler , B. Bolker , and S. Walker . 2015. “Fitting Linear Mixed‐Effects Models Using lme4.” Journal of Statistical Software 67(1): 1–48. 10.18637/jss.v067.i01.

[eap70180-bib-0007] Beaune, D. , B. Fruth , L. Bollache , G. Hohmann , and F. Bretagnolle . 2013. “Doom of the Elephant‐Dependent Trees in a Congo Tropical Forest.” Forest Ecology and Management 295: 109–117. 10.1016/j.foreco.2012.12.041.

[eap70180-bib-0008] Beck, H. , J. W. Snodgrass , and P. Thebpanya . 2013. “Long‐Term Exclosure of Large Terrestrial Vertebrates: Implications of Defaunation for Seedling Demographics in the Amazon Rainforest.” Biological Conservation 163: 115–121. 10.1016/j.biocon.2013.03.012.

[eap70180-bib-0009] Beckman, N. G. , and H. S. Rogers . 2013. “Consequences of Seed Dispersal for Plant Recruitment in Tropical Forests: Interactions within the Seedscape.” Biotropica 45: 666–681. 10.1111/btp.12071.

[eap70180-bib-0011] Benitez, L. , and S. A. Queenborough . 2021. “Fruit Trees Drive Small‐Scale Movement of Elephants in Kibale National Park, Uganda.” Biotropica 53: 1620–1630. 10.1111/btp.13010.

[eap70180-bib-0012] Berzaghi, F. , F. Bretagnolle , C. Durand‐Bessart , and S. Blake . 2023. “Megaherbivores Modify Forest Structure and Increase Carbon Stocks through Multiple Pathways.” Proceedings of the National Academy of Sciences 120: e2201832120. 10.1073/pnas.2201832120. PMC9945987 36689651

[eap70180-bib-0013] Blake, S. , and F. Maisels . 2023. “Forest Elephant Movements in Central Africa: Megafauna Need Megaspaces.” In Movement Ecology of Afrotropical Forest Mammals, edited by R. Reyna‐Hurtado , C. A. Chapman , and M. Melletti , 27–58. Cham: Springer International Publishing. 10.1007/978-3-031-27030-7_3.

[eap70180-bib-0014] Blake, S. , S. Strindberg , P. Boudjan , C. Makombo , I. Bila‐Isia , O. Ilambu , F. Grossmann , et al. 2007. “Forest Elephant Crisis in The Congo Basin.” PLoS Biology 5: e111. 10.1371/journal.pbio.0050111. 17407383 PMC1845159

[eap70180-bib-0015] Blonder, B. , S. Both , D. A. Coomes , D. Elias , T. Jucker , J. Kvasnica , N. Majalap , et al. 2018. “Extreme and Highly Heterogeneous Microclimates in Selectively Logged Tropical Forests.” Frontiers in Forests and Global Change 1, 5. 10.3389/ffgc.2018.00005.

[eap70180-bib-0016] Brugière, D. , S. Bougras , and A. Gautier‐Hion . 2000. Dynamique forestière à processus de colonisation‐extinction: relations faune flore dans les forêts à Marantacées du Parc National d'Odzala 1–10. Bruxelles, Belgium: Rapport ECOFAC.

[eap70180-bib-0017] Burak, M. K. , K. M. Ferraro , K. D. Orrick , N. R. Sommer , D. Ellis‐Soto , and O. J. Schmitz . 2024. “Context Matters when Rewilding for Climate Change.” People and Nature 6: 507–518. 10.1002/pan3.10609.

[eap70180-bib-0018] Burivalova, Z. , S. Orndorff , A. Truskinger , P. Roe , and E. T. Game . 2021. “The Sound of Logging: Tropical Forest Soundscape Before, during, and after Selective Timber Extraction.” Biological Conservation 254: 108812. 10.1016/j.biocon.2020.108812.

[eap70180-bib-0019] Burivalova, Z. , Ç. H. Şekercioğlu , and L. P. Koh . 2014. “Thresholds of Logging Intensity to Maintain Tropical Forest Biodiversity.” Current Biology 24: 1893–1898. 10.1016/j.cub.2014.06.065. 25088557

[eap70180-bib-0020] Campos‐Arceiz, A. , and S. Blake . 2011. “Megagardeners of the Forest – The Role of Elephants in Seed Dispersal.” Acta Oecologica 37: 542–553. 10.1016/j.actao.2011.01.014.

[eap70180-bib-0022] Cardoso, A. W. , Y. Malhi , I. Oliveras , D. Lehmann , J. E. Ndong , E. Dimoto , E. Bush , et al. 2020. “The Role of Forest Elephants in Shaping Tropical Forest–Savanna Coexistence.” Ecosystems 23(3): 602–616. 10.1007/s10021-019-00424-3.

[eap70180-bib-0024] CBD . 2018. “United Nations Convention on Biological Diversity: Protected Areas and Other Effective Area‐Based Conservation Measures.” *CBD/COP/DEC/14/8*. 30 November 2018 https://www.cbd.int/doc/decisions/cop-14/cop-14-dec-08-en.pdf

[eap70180-bib-0025] Chapman, C. A. , and L. J. Chapman . 1995. “Survival without Dispersers: Seedling Recruitment under Parents.” Conservation Biology 9: 675–678.

[eap70180-bib-0026] Chaudhary, A. , Z. Burivalova , L. P. Koh , and S. Hellweg . 2016. “Impact of Forest Management on Species Richness: Global Meta‐Analysis and Economic Trade‐Offs.” Scientific Reports 6: 23954. 10.1038/srep23954. 27040604 PMC4819217

[eap70180-bib-0027] Chazdon, R. L. 1988. “Sunflecks and their Importance to Forest Understorey Plants.” Advances in Ecological Research, 18, 1–63. 10.1016/S0065-2504(08)60179-8.

[eap70180-bib-0028] Clark, C. J. , J. R. Poulsen , R. Malonga , and P. W. Elkan . 2009. “Logging Concessions Can Extend the Conservation Estate for Central African Tropical Forests.” Conservation Biology 23: 1281–1293. 10.1111/j.1523-1739.2009.01243.x. 19453655

[eap70180-bib-0029] Condit, R. , R. Pérez , S. Lao , S. Aguilar , and S. P. Hubbell . 2017. “Demographic Trends and Climate over 35 Years in the Barro Colorado 50 ha Plot.” Forest Ecosystems 4: 17. 10.1186/s40663-017-0103-1.

[eap70180-bib-0030] Culot, L. , C. Bello , J. L. F. Batista , H. T. Z. do Couto , and M. Galetti . 2017. “Synergistic Effects of Seed Disperser and Predator Loss on Recruitment Success and Long‐Term Consequences for Carbon Stocks in Tropical Rainforests.” Scientific Reports 7: 7662. 10.1038/s41598-017-08222-4. 28794422 PMC5550475

[eap70180-bib-0031] de Carvalho, A. L. , M. V. N. d'Oliveira , F. E. Putz , and L. C. de Oliveira . 2017. “Natural Regeneration of Trees in Selectively Logged Forest in Western Amazonia.” Forest Ecology and Management 392: 36–44. 10.1016/j.foreco.2017.02.049.

[eap70180-bib-0032] Denslow, J. S. , J. C. Schultz , P. M. Vitousek , and B. R. Strain . 1990. “Growth Responses of Tropical Shrubs to Treefall Gap Environments.” Ecology 71: 165–179. 10.2307/1940257.

[eap70180-bib-0033] Duah‐Gyamfi, A. , B. Kyereh , K. A. Adam , V. K. Agyeman , and M. D. Swaine . 2014. “Natural Regeneration Dynamics of Tree Seedlings on Skid Trails and Tree Gaps Following Selective Logging in a Tropical Moist Semi‐Deciduous Forest in Ghana.” Open Journal of Forestry 4: 49–57. 10.4236/ojf.2014.41009.

[eap70180-bib-0034] Duvall, E. S. , E. le Roux , H. C. Pearson , J. Roman , Y. Malhi , and A. J. Abraham . 2024. “Resisting the Carbonization of Animals as Climate Solutions.” Nature Climate Change 14: 892–895. 10.1038/s41558-024-02106-y.

[eap70180-bib-0035] EDF . 2023. HFLD Factsheet: What Are HFLDS? And why Are they Important? New York, NY: Environmental Defense Fund https://www.edf.org/sites/default/files/2023-03/HFLD%20factsheet_final.pdf.

[eap70180-bib-0036] Ellis, P. W. , T. Gopalakrishna , R. C. Goodman , F. E. Putz , A. Roopsind , P. M. Umunay , J. Zalman , et al. 2019. “Reduced‐Impact Logging for Climate Change Mitigation (RIL‐C) Can Halve Selective Logging Emissions from Tropical Forests.” Forest Ecology and Management 438: 255–266. 10.1016/j.foreco.2019.02.004.

[eap70180-bib-0037] Fayolle, A. , D.‐Y. Ouédraogo , G. Ligot , K. Daïnou , N. Bourland , P. Tekam , and J.‐L. Doucet . 2015. “Differential Performance between Two Timber Species in Forest Logging Gaps and in Plantations in Central Africa.” Forests 6: 380–394. 10.3390/f6020380.

[eap70180-bib-0038] Forget, P. M. , and D. Wenny . 2005. “How to Elucidate Seed Fate? A Review of Methods Used to Study Seed Removal and Secondary Seed Dispersal. Seed Fate: Predation, Dispersal and Seedling Establishment.” In Seed Fate: Predation, Dispersal and Seedling Establishment Oxfordshire: CABI Publishing. 379–394.

[eap70180-bib-0039] FSC . 2023. “Annual Report 2023.” https://fsc.org/en/media/fsc-annual-report-2023

[eap70180-bib-0040] Gillet, J.‐F. 2013. “Les forêts à Marantaceae au sein de la mosaïque forestière du Nord de la République du Congo: Origines et modalités de gestion.” PhD dissertation, Université de Liège‐Gembloux Agro‐Bio Tech, Gembloux, Belgium.

[eap70180-bib-0041] Gourlet‐Fleury, S. , F. Mortier , A. Fayolle , F. Baya , D. Ouédraogo , F. Bénédet , and N. Picard . 2013. “Tropical Forest Recovery from Logging: A 24 Year Silvicultural Experiment from Central Africa.” Philosophical Transactions of the Royal Society B: Biological Sciences 368: 20120302. 10.1098/rstb.2012.0302. PMC3720023 23878332

[eap70180-bib-0042] Hartig, F. 2024. “DHARMa: Residual Diagnostics for Hierarchical (Multi‐Level/Mixed) Regression Models.” R package version 0.4.7, https://CRAN.R-project.org/package=DHARMa.

[eap70180-bib-0043] Hockridge, E. G. , E. M. Bradford , K. I. W. Angier , B. H. Youd , E. B. M. McGill , S. Y. Ngouma , R. L. Ognangue , G. E. M. Gibbon , and A. B. Davies . 2024. “Spatial Ecology, Biodiversity, and Abiotic Determinants of Congo's Bai Ecosystem.” Ecology 105(11): e4419. 10.1002/ecy.4419. 39352298

[eap70180-bib-0046] ITTO . 2024. “Lighting the Path to Sustainable Development.” In Tropical Forest Update. Yokohama: ITTO. https://www.itto.int/direct/topics/topics_pdf_download/topics_id=7951&no=1&disp=inline.

[eap70180-bib-0048] John, J. A. , and N. R. Draper . 1980. “An Alternative Family of Transformations.” Journal of the Royal Statistical Society. Series C (Applied Statistics) 29: 190. 10.2307/2986305.

[eap70180-bib-0049] Karsenty, A. 2016. “The Contemporary Forest Concessions in West and Central Africa: Chronicle of a Foretold Decline?” In FAO Forest Policy and Institutions Working Paper 34. Rome: Food and Agriculture Organization of the United Nations. https://agritrop.cirad.fr/581423/1/Africa%20Report_Karsenty_12%2008%2016.pdf.

[eap70180-bib-0050] Keany, J. M. , P. Burns , A. J. Abraham , P. Jantz , L. Makaga , S. Saatchi , F. Maisels , K. Abernethy , and C. E. Doughty . 2024. “Using Multiscale Lidar to Determine Variation in Canopy Structure from African Forest Elephant Trails.” Remote Sensing in Ecology and Conservation 10: 655–667. 10.1002/rse2.395.

[eap70180-bib-0051] Kleinschroth, F. , and J. R. Healey . 2017. “Impacts of Logging Roads on Tropical Forests.” Biotropica 49: 620–635. 10.1111/btp.12462.

[eap70180-bib-0053] Lawes, M. J. , and C. A. Chapman . 2006. “Does the Herb *Acanthus pubescens* and/or Elephants Suppress Tree Regeneration in Disturbed Afrotropical Forest?” Forest Ecology and Management 221: 278–284. 10.1016/j.foreco.2005.10.039.

[eap70180-bib-0054] Lemmon, P. E. 1956. “A Spherical Densiometer for Estimating Forest Overstory Density.” Forest 2: 314–320.

[eap70180-bib-0055] Lescuyer, G. , R. Tsanga , S. Nziengui , E. Forni , and C. Romero . 2021. “Influence of FSC Certification on the Governance of the Logging Sector in The Congo Basin.” Natural Resources Forum 45: 289–304. 10.1111/1477-8947.12231.

[eap70180-bib-0056] Levis, C. , F. R. C. Costa , F. Bongers , M. Peña‐Claros , C. R. Clement , A. B. Junqueira , E. G. Neves , et al. 2017. “Persistent Effects of Pre‐Columbian Plant Domestication on Amazonian Forest Composition.” Science 355(6328): 925–931. 28254935 10.1126/science.aal0157

[eap70180-bib-0057] Lhoest, S. , D. Fonteyn , K. Daïnou , L. Delbeke , J.‐L. Doucet , M. Dufrêne , J.‐F. Josso , et al. 2020. “Conservation Value of Tropical Forests: Distance to Human Settlements Matters More than Management in Central Africa.” Biological Conservation 241: 108351. 10.1016/j.biocon.2019.108351.

[eap70180-bib-0058] Lowe, R. G. , and P. Walker . 1977. “Classification of Canopy, Stem, Crown Status and Climber Infestation in Natural Tropical Forest in Nigeria.” Journal of Applied Ecology 14: 897–903. 10.2307/2402820.

[eap70180-bib-0059] Lüdecke, D. , M. S. Ben‐Shachar , I. Patil , P. Waggoner , and D. Makowski . 2021. “performance: An R Package for Assessment, Comparison and Testing of Statistical Models.” Journal of Open Source Software 6: 3139. 10.21105/joss.03139.

[eap70180-bib-0060] Maisels, F. , S. Strindberg , S. Blake , G. Wittemyer , J. Hart , E. A. Williamson , R. Aba'a , et al. 2013. “Devastating Decline of Forest Elephants in Central Africa.” PLoS One 8: e59469. 10.1371/journal.pone.0059469. 23469289 PMC3587600

[eap70180-bib-0061] Malhi, Y. , C. Girardin , D. B. Metcalfe , C. E. Doughty , L. E. O. C. Aragão , S. W. Rifai , I. Oliveras , et al. 2021. “The Global Ecosystems Monitoring Network: Monitoring Ecosystem Productivity and Carbon Cycling across the Tropics.” Biological Conservation 253: 108889. 10.1016/j.biocon.2020.108889.

[eap70180-bib-0062] Malhi, Y. , T. Riutta , O. R. Wearn , N. J. Deere , S. L. Mitchell , H. Bernard , N. Majalap , et al. 2022. “Logged Tropical Forests Have Amplified and Diverse Ecosystem Energetics.” Nature 612: 707–713. 10.1038/s41586-022-05523-1. 36517596 PMC9771799

[eap70180-bib-0063] Medjibe, V. P. , F. E. Putz , M. P. Starkey , A. A. Ndouna , and H. R. Memiaghe . 2011. “Impacts of Selective Logging on above‐Ground Forest Biomass in the Monts de Cristal in Gabon.” Forest Ecology and Management 262: 1799–1806. 10.1016/j.foreco.2011.07.014.

[eap70180-bib-0064] Merz, G. 1981. “Research on the Nutrition Biology and on the Habitat Preferences of the Forest Elephant, Loxodonta‐Africana‐Cyclotis Matschie, 1900.” Mammalia 45: 299–312.

[eap70180-bib-0065] Mills, M. B. , Y. Malhi , R. M. Ewers , L. K. Kho , Y. A. Teh , S. Both , D. F. R. P. Burslem , et al. 2023. “Tropical Forests Post‐Logging Are a Persistent Net Carbon Source to the Atmosphere.” Proceedings of the National Academy of Sciences 120: e2214462120. 10.1073/pnas.2214462120. PMC9934015 36623189

[eap70180-bib-0066] Morin‐Rivat, J. , A. Fayolle , C. Favier , L. Bremond , S. Gourlet‐Fleury , N. Bayol , P. Lejeune , H. Beeckman , and J.‐L. Doucet . 2017. “Present‐Day Central African Forest Is a Legacy of the 19th Century Human History.” eLife 6: e20343. 10.7554/eLife.20343. 28093097 PMC5241113

[eap70180-bib-0067] Omeja, P. A. , M. J. Lawes , A. Corriveau , K. Valenta , D. Sarkar , F. P. Paim , and C. A. Chapman . 2016. “Recovery of Tree and Mammal Communities during Large‐Scale Forest Regeneration in Kibale National Park, Uganda.” Biotropica 48: 770–779. 10.1111/btp.12360.

[eap70180-bib-0068] Osazuwa‐Peters, O. L. , C. A. Chapman , and A. E. Zanne . 2015. “Selective Logging: Does the Imprint Remain on Tree Structure and Composition after 45 Years?” Conservation Physiology 3: cov012. 10.1093/conphys/cov012. 27293697 PMC4778436

[eap70180-bib-0069] Paul, J. R. , A. M. Randle , C. A. Chapman , and L. J. Chapman . 2004. “Arrested Succession in Logging Gaps: Is Tree Seedling Growth and Survival Limiting?” African Journal of Ecology 42: 245–251. 10.1111/j.1365-2028.2004.00435.x.

[eap70180-bib-0070] Pfeifer, M. , V. Lefebvre , E. Turner , J. Cusack , M. Khoo , V. K. Chey , M. Peni , and R. M. Ewers . 2015. “Deadwood Biomass: An Underestimated Carbon Stock in Degraded Tropical Forests?” Environmental Research Letters 10: 044019. 10.1088/1748-9326/10/4/044019.

[eap70180-bib-0071] Phillips, O. L. , and A. H. Gentry . 1994. “Increasing Turnover through Time in Tropical Forests.” Science 263: 954–958. 10.1126/science.263.5149.954. 17758638

[eap70180-bib-0072] Picard, J. 2024. “The spatio‐temporal dynamics of Marantaceae forests in Central Africa.” PhD thesis, Université de Montpellier, https://theses.hal.science/tel-05125497

[eap70180-bib-0073] Piiroinen, T. , A. Valtonen , and H. Roininen . 2017. “Vertebrate Herbivores Are the Main Cause of Seedling Mortality in a Logged African Rainforest—Implications for Forest Restoration.” Restoration Ecology 25: 442–452. 10.1111/rec.12460.

[eap70180-bib-0074] Pillay, R. , F. Hua , B. A. Loiselle , H. Bernard , and R. J. Fletcher . 2018. “Multiple Stages of Tree Seedling Recruitment Are Altered in Tropical Forests Degraded by Selective Logging.” Ecology and Evolution 8: 8231–8242. 10.1002/ece3.4352. 30250698 PMC6145000

[eap70180-bib-0075] Poulsen, J. R. , C. J. Clark , and B. M. Bolker . 2011. “Decoupling the Effects of Logging and Hunting on an Afrotropical Animal Community.” Ecological Applications 21: 1819–1836. 10.1890/10-1083.1. 21830721

[eap70180-bib-0076] Pouteau, R. , J. Picard , C. Doumenge , T. Brncic , J.‐F. Gillet , J.‐L. Doucet , S. Gourlet‐Fleury , et al. 2024. “The Puzzling Ecology of African Marantaceae Forests.” American Journal of Botany 111: e16320. 10.1002/ajb2.16320. 38629307

[eap70180-bib-0077] Putz, F. E. 1984. “The Natural History of Lianas on Barro Colorado Island, Panama.” Ecology 65: 1713–1724. 10.2307/1937767.

[eap70180-bib-0078] Putz, F. E. , T. Baker , B. W. Griscom , T. Gopalakrishna , A. Roopsind , P. M. Umunay , J. Zalman , E. A. Ellis , Ruslandi , and P. W. Ellis . 2019. “Intact Forest in Selective Logging Landscapes in the Tropics.” Frontiers in Forests and Global Change 2, 30. 10.3389/ffgc.2019.00030

[eap70180-bib-0079] Putz, F. E. , P. A. Zuidema , T. Synnott , M. Peña‐Claros , M. A. Pinard , D. Sheil , J. K. Vanclay , et al. 2012. “Sustaining Conservation Values in Selectively Logged Tropical Forests: The Attained and the Attainable.” Conservation Letters 5: 296–303. 10.1111/j.1755-263X.2012.00242.x.

[eap70180-bib-0080] Rocha, E. X. , J. Schietti , C. S. Gerolamo , R. J. Burnham , and A. Nogueira . 2020. “Higher Rates of Liana Regeneration after Canopy Fall Drives Species Abundance Patterns in Central Amazonia.” Journal of Ecology 108: 1311–1321. 10.1111/1365-2745.13345.

[eap70180-bib-0081] Romero, C. , E. O. Sills , M. R. Guariguata , P. O. Cerutti , G. Lescuyer , and F. E. Putz . 2017. “Evaluation of the Impacts of Forest Stewardship Council (FSC) Certification of Natural Forest Management in the Tropics: A Rigorous Approach to Assessment of a Complex Conservation Intervention.” International Forestry Review 19: 36–49. 10.1505/146554817822295902.

[eap70180-bib-0082] Roopsind, A. , T. T. Caughlin , P. van der Hout , E. Arets , and F. E. Putz . 2018. “Trade‐Offs between Carbon Stocks and Timber Recovery in Tropical Forests Are Mediated by Logging Intensity.” Global Change Biology 24: 2862–2874. 10.1111/gcb.14155. 29603495

[eap70180-bib-0083] Rosin, C. , K. K. Beals , M. W. Belovitch , R. E. Harrison , M. Pendred , M. K. Sullivan , N. Yao , and J. R. Poulsen . 2020. “Assessing the Effects of Elephant Foraging on the Structure and Diversity of an Afrotropical Forest.” Biotropica 52: 502–508. 10.1111/btp.12758.

[eap70180-bib-0085] Rosin, C. , J. R. Poulsen , V. Swamy , and A. Granados . 2017. “A Pantropical Assessment of Vertebrate Physical Damage to Forest Seedlings and the Effects of Defaunation.” Global Ecology and Conservation 11: 188–195. 10.1016/j.gecco.2017.06.001.

[eap70180-bib-0086] Rueda‐Trujillo, M. A. , M. P. Veldhuis , P. M. van Bodegom , H. P. T. de Deurwaerder , and M. Visser . 2024. “Global Increase of Lianas in Tropical Forests.” Global Change Biology 30: e17485. 10.1111/gcb.17485. 39187993

[eap70180-bib-0087] Runting, R. K. , Ruslandi , B. W. Griscom , M. J. Struebig , M. Satar , E. Meijaard , Z. Burivalova , et al. 2019. “Larger Gains from Improved Management over Sparing–Sharing for Tropical Forests.” Nature Sustainability 2: 53–61. 10.1038/s41893-018-0203-0.

[eap70180-bib-0088] Scalbert, M. , D. Fonteyn , F. Houngbégnon , R. Scalbert , C. Vermeulen , B. Haurez , S. Lhoest , et al. 2025. “Short‐Term Impacts of Selective Logging on Forest Elephants.” Conservation Science and Practice 7: e13300. 10.1111/csp2.13300.

[eap70180-bib-0089] Scalbert, M. , Q. Stiernon , S. Franceschini , C. Vermeulen , Y. Brostaux , R. Ngwet , and J.‐L. Doucet . 2023. “Not all Roads Are Barriers: Large Mammals Use Logging Roads in a Timber Concession of South‐Eastern Cameroon.” Forest Ecology and Management 541: 120910. 10.1016/j.foreco.2023.120910.

[eap70180-bib-0090] Scalbert, M. , C. Vermeulen , T. Breuer , and J.‐L. Doucet . 2023. “The Challenging Coexistence of Forest Elephants Loxodonta Cyclotis and Timber Concessions in Central Africa.” Mammal Review 53: 15–31. 10.1111/mam.12305.

[eap70180-bib-0091] Schnitzer, S. A. , and F. Bongers . 2011. “Increasing Liana Abundance and Biomass in Tropical Forests: Emerging Patterns and Putative Mechanisms.” Ecology Letters 14: 397–406. 10.1111/j.1461-0248.2011.01590.x. 21314879

[eap70180-bib-0092] Schnitzer, S. A. , D. M. DeFilippis , M. Visser , S. Estrada‐Villegas , R. Rivera‐Camaña , B. Bernal , S. Peréz , et al. 2021. “Local Canopy Disturbance as an Explanation for Long‐Term Increases in Liana Abundance.” Ecology Letters 24: 2635–2647. 10.1111/ele.13881. 34536250

[eap70180-bib-0093] Schnitzer, S. A. , S. Estrada‐Villegas , and S. J. Wright . 2020. “The Response of Lianas to 20 yr of Nutrient Addition in a Panamanian Forest.” Ecology 101: e03190. 10.1002/ecy.3190. 32893876

[eap70180-bib-0094] SEEF . 2019. “Bilan d'exploitation AAC 2018.” (5398 ha) UFG2 – UFA2 NZAMALIGUE. *SEEF Report*

[eap70180-bib-0095] Short, J. 1981. “Diet and Feeding Behaviour of the Forest Elephant.” Diet and Feeding Behaviour of the Forest Elephant 45: 177–186. 10.1515/mamm.1981.45.2.177.

[eap70180-bib-0096] Sist, P. 2000. “Reduced‐Impact Logging in the Tropics : Objectives, Principles and Impacts.” The International Forestry Review 2: 3–10.

[eap70180-bib-0097] Stokes, E. J. , S. Strindberg , P. C. Bakabana , P. W. Elkan , F. C. Iyenguet , B. Madzoké , G. A. F. Malanda , et al. 2010. “Monitoring Great Ape and Elephant Abundance at Large Spatial Scales: Measuring Effectiveness of a Conservation Landscape.” PLoS One 5: e10294. 10.1371/journal.pone.0010294. 20428233 PMC2859051

[eap70180-bib-0098] Struhsaker, T. T. , J. S. Lwanga , and J. M. Kasenene . 1996. “Elephants, Selective Logging and Forest Regeneration in the Kibale Forest, Uganda.” Journal of Tropical Ecology 12: 45–64. 10.1017/S0266467400009305.

[eap70180-bib-0099] Sullivan, M. K. , P. A. M. Biessiemou , R. Niangadouma , K. Abernethy , S. A. Queenborough , and L. Comita . 2022. “A Decade of Diversity and Forest Structure: Post‐Logging Patterns across Life Stages in an Afrotropical Forest.” Forest Ecology and Management 513: 120169. 10.1016/j.foreco.2022.120169.

[eap70180-bib-0100] Sullivan, M. K. , L. Browne , P. A. M. Bissiemou , R. Niangadouma , K. Abernethy , S. Queenborough , and L. S. Comita . 2025. “Data from: Timber and Trails: Low‐intensity Selective Logging and Elephant Trails Shape Seedling Dynamics in An Afrotropical Forest [Dataset].” Dryad. 10.5061/dryad.zs7h44jkp 41622836

[eap70180-bib-0101] Sullivan, M. K. , J. Vleminckx , P. A. M. Bissiemou , R. Niangadouma , M. I. Mayoungou , J. L. Temba , F. Bénédet , K. Abernethy , S. A. Queenborough , and L. S. Comita . 2024. “Low‐Intensity Logging Alters Species and Functional Composition, but Does Not Negatively Impact Key Ecosystem Services in a Central African Tropical Forest.” Global Ecology and Conservation 53: e02996. 10.1016/j.gecco.2024.e02996.

[eap70180-bib-0102] Sunderland, T. , G. M. Walters , and Y. Issembe . 2004. “A Preliminary Vegetation Assessment of the Mbé National Park, Monts de Cristal, Gabon.” Central African Regional Program for the Environment (CARPE), 1–51. 10.13140/2.1.2193.6321.

[eap70180-bib-0103] Terborgh, J. , and S. J. Wright . 1994. “Effects of Mammalian Herbivores on Plant Recruitment in Two Neotropical Forests.” Ecology 75: 1829–1833. 10.2307/1939641.

[eap70180-bib-0113] Turkalo, A. K. , P. H. Wrege , and G. Wittemyer . 2013. “Long‐Term Monitoring of Dzanga Bai Forest Elephants: Forest Clearing use Patterns.” PloS one 8: e85154. 24386460 10.1371/journal.pone.0085154 PMC3873458

[eap70180-bib-0104] Umunay, P. M. , T. G. Gregoire , T. Gopalakrishna , P. W. Ellis , and F. E. Putz . 2019. “Selective Logging Emissions and Potential Emission Reductions from Reduced‐Impact Logging in The Congo Basin.” Forest Ecology and Management 437: 360–371. 10.1016/j.foreco.2019.01.049.

[eap70180-bib-0105] van Vliet, N. , and R. Nasi . 2008. “Hunting for Livelihood in Northeast Gabon: Patterns, Evolution, and Sustainability.” Ecology and Society 13: art33.

[eap70180-bib-0106] Vande Weghe, J. P. 2008. Monts de Cristal: les Parcs nationaux du Gabon. New York: Wildlife Conservation Society (WCS).

[eap70180-bib-0107] White, L. J. T. , M. E. Rogers , C. E. G. Tutin , E. A. Williamson , and M. Fernandez . 1995. “Herbaceous Vegetation in Different Forest Types in the Lopé Reserve, Gabon: Implications for Keystone Food Availability.” African Journal of Ecology 33: 124–141. 10.1111/j.1365-2028.1995.tb00788.x.

[eap70180-bib-0108] Whitmore, T. C. 1989. “Canopy Gaps and the Two Major Groups of Forest Trees.” Ecology 70: 536–538. 10.2307/1940195.

[eap70180-bib-0109] Wright, S. J. 2002. “Plant Diversity in Tropical Forests: A Review of Mechanisms of Species Coexistence.” Oecologia 130: 1–14. 10.1007/s004420100809. 28547014

[eap70180-bib-0110] Yamada, T. , A. Yoshioka , M. Hashim , N. Liang , and T. Okuda . 2014. “Spatial and Temporal Variations in the Light Environment in a Primary and Selectively Logged Forest Long after Logging in Peninsular Malaysia.” Trees 28: 1355–1365. 10.1007/s00468-014-1040-z.

[eap70180-bib-0111] Yoh, N. , W. Mbamy , B. L. Gottesman , G. Z. L. Froese , T. Satchivi , M. Obiang Ebanega , L. Carlson , et al. 2024. “Impacts of Logging, Hunting, and Conservation on Vocalizing Biodiversity in Gabon.” Biological Conservation 296: 110726. 10.1016/j.biocon.2024.110726.

[eap70180-bib-0112] Zwerts, J. A. , E. H. M. Sterck , P. A. Verweij , F. Maisels , J. van der Waarde , E. A. M. Geelen , G. B. Tchoumba , H. F. Donfouet Zebaze , and M. van Kuijk . 2024. “FSC‐Certified Forest Management Benefits Large Mammals Compared to Non‐FSC.” Nature 628: 563–568. 10.1038/s41586-024-07257-8. 38600379 PMC11023928

