# Supplementary material for: Timber and trails: Low‐intensity selective logging and elephant trails shape seedling dynamics in an Afrotropical forest
Source: Ecol Appl. 2026 Feb 1;36(1):e70180. doi: 10.1002/eap.70180 (PMC12862287; doi:10.1002/eap.70180)
Supplement: Supplementary file 1 — Appendix S1: [file EAP-36-e70180-s001.pdf]

Appendix S1

Timber and trails: Low-intensity selective logging and elephant trails shape seedling dynamics in an Afrotropical forest

Megan K. Sullivan, Luke Browne, Prince Armel Mouguiama Bissiemou, Raoul Niangadouma,

Katharine Abernethy, Simon A. Queenborough, Liza S. Comita

Ecological Applications

Logging treatment differences

Environmental factors during and after selective logging

	(a) canopy openness			(b) damage			(c) trails		
Predictors	*Incidence Rate Ratios*	*CI*	*p*	*Odds Ratios*	*CI*	*p*	*Odds Ratios*	*CI*	*p*
(Intercept)	7.67	6.93 – 8.47	**<0.001**	0.15	0.08 – 0.26	**<0.001**	0.50	0.34 – 0.78	**0.002**
plot type3 [logged.2008]	0.97	0.84 – 1.11	0.627	1.84	0.86 – 3.93	0.116	2.09	1.18 – 3.72	**0.012**
plot type3 [logged.2018]	1.26	1.10 – 1.46	**0.001**	4.59	2.18 – 9.65	**<0.001**	1.45	0.83 – 2.55	0.191
plot type3 [logged.2020]	1.16	1.00 – 1.33	**0.043**	7.26	3.47 – 15.21	**<0.001**	0.69	0.39 – 1.23	0.207
**Random Effects**									
σ^2	0.19			3.29			3.29		
τ_{00}	0.03 $_{plot}$			0.68 $_{plot}$			0.31 $_{plot}$		
ICC	0.13			0.17			0.09		
N	80 $_{plot}$			80 $_{plot}$			80 $_{plot}$		
Observations	720			720			720		
Marginal R^2 / Conditional R^2	0.052 / 0.178			0.133 / 0.280			0.045 / 0.128		

Table S1. Differences in (a) canopy openness in subplots, (b) presence/absence of logging and vegetation damage in subplots, (a) presence/absence of elephant trails in subplots and across the logging chronosequence. Linear mixed-effects models were used to test whether environmental factors were significantly different for subplots in each of the three logging treatments compared to unlogged forests. Random effects show the residual variance (σ^2), plot-level variance (τ_{00}), intraclass correlation coefficient (ICC), the number of plots (N), total observations, and the variance explained by fixed effects (Marginal R^2) and the full model (Conditional R^2).

Figure S1.

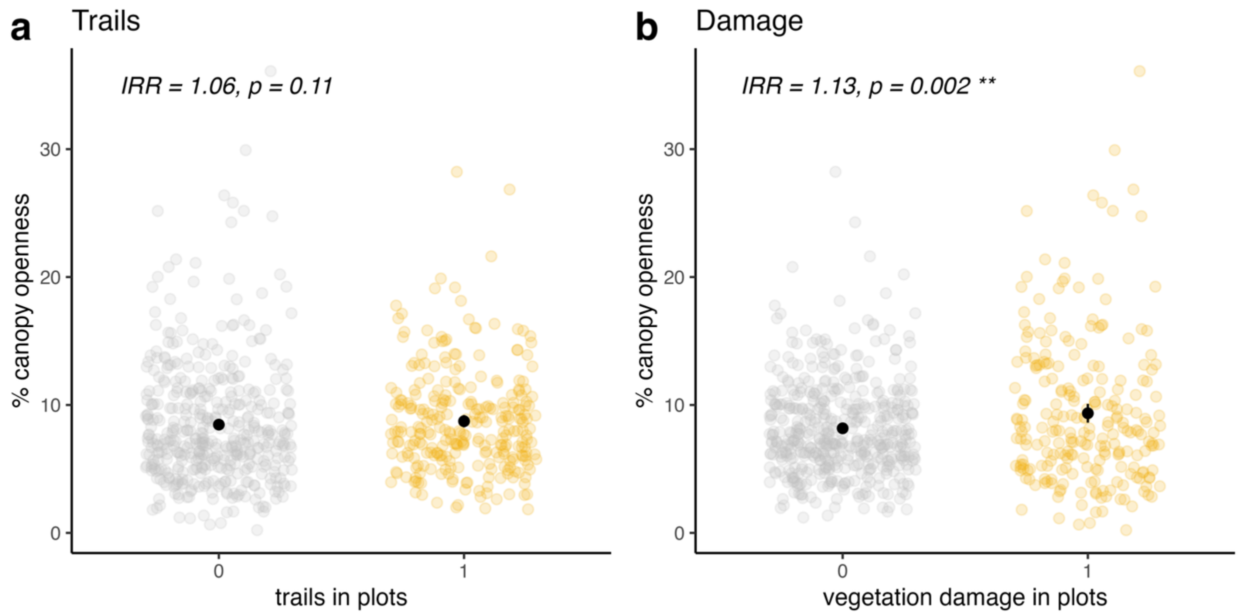

Figure S1: Differences in (a) canopy openness in plots with elephant trails, (b) canopy openness in plots with vegetation damage. Points refer to presence (1) or absence (0) of vegetation damage in plot (a), and presence (1) or absence (0) of elephant trails in plot (b). Boxes represent inter-quartile range (IQR), lines in the center represent the median. Whiskers correspond to the largest/smallest value less/greater than upper/lower quartile minus 1.5 times IQR. ** denotes significant differences at the P<0.01 levels.

Canopy openness differences on trails and with vegetation damage

	(a) trails – canopy openness			(b) damage – canopy openness		
Predictors	*Incidence Rate Ratios*	*CI*	*p*	*Incidence Rate Ratios*	*CI*	*p*
(Intercept)	8.12	7.66 – 8.69	**<0.001**	8.02	7.54 – 8.52	**<0.001**
trails	1.06	0.99 – 1.14	0.108			
damage				1.13	1.05 – 1.22	**0.002**
**Random Effects**						
σ^2	0.19			0.19		
τ_{00}	0.04 $_{plot}$			0.04 $_{plot}$		
ICC	0.18			0.17		
N	80 $_{plot}$			80 $_{plot}$		
Observations	720			720		
Marginal R^2/ Conditional R^2	0.004 / 0.186			0.015 / 0.187		

Table S2:. Linear mixed-effects models were used to test whether canopy openness levels were significantly different in (a) subplots with elephant trails vs. without elephant trails and (b) subplots with logging and vegetation damage vs. without logging and vegetation damage. Random effects show the residual variance (σ^2), plot-level variance (τ_{00}), intraclass correlation coefficient (ICC), the number of plots (N), total observations, and the variance explained by fixed effects (Marginal R^2) and the full model (Conditional R^2).

Seedling performance models – environmental factors

Environmental factors

	(a) survival			(b) growth			(c) recruitment		
Predictors	*Risk Ratios*	*CI*	*p*	*Estimates*	*CI*	*p*	*Incidence Rate Ratios*	*CI*	*p*
(Intercept)	10.73	4.34 – 26.53	**<0.001**	0.69	0.64 – 0.74	**<0.001**	0.93	0.74 – 1.17	0.514
height (last census)	1.49	1.35 – 1.64	**<0.001**	-0.15	-0.16 – -0.15	**<0.001**			
canopy openness	0.98	0.96 – 1.00	**0.023**	0.00	-0.00 – 0.00	0.130	1.00	0.98 – 1.02	0.696
damage	0.78	0.65 – 0.93	**0.007**	0.01	-0.00 – 0.03	0.103	0.94	0.77 – 1.14	0.543
trail	0.67	0.57 – 0.80	**<0.001**	-0.01	-0.03 – 0.00	0.103	1.07	0.90 – 1.28	0.432
**Random Effects**									
σ^2	1.64			0.01			0.84		
τ_{00}	0.16 $_{plot.sub}$			0.00 $_{plot.sub}$			0.17 $_{plot}$		
	0.34 $_{plot}$			0.00 $_{plot}$					
	16.15 $_{spp3}$			0.01 $_{spp3}$					
ICC	0.91			0.51			0.17		
N	71 $_{spp3}$			68 $_{spp3}$			80 $_{plot}$		
	80 $_{plot}$			79 $_{plot}$					
	645 $_{plot.sub}$			553 $_{plot.sub}$					
Observations	2251			1595			720		
Marginal R^2 / Conditional R^2	0.008 / 0.911			0.331 / 0.675			0.003 / 0.173		
AIC	2313.014			-2202.655			2173.451		

Table S3: Differences in (a) survival, (b) growth, and (c) recruitment as a function of the presence/absence of elephant trails in subplots. Linear mixed-effects models were used to test how different environmental factors influenced seedling performance. Statistics are from full generalized linear models (i.e. all three predictors were included in each of the survival, growth, and recruitment models). Random effects show the residual variance (σ^2), plot-level variance (τ_{00}), intraclass correlation coefficient (ICC), the number of plots (N), total observations, and the variance explained by fixed effects (Marginal R^2) and the full model (Conditional R^2).

Check Collinearity

Term	*VIF*	*VIF_CI_low*	*VIF_CI_high*	*SE_factor*	*Tolerance*	*Tolerance _CI_low*	*Tolerance _CI_high*
**(a) survival**							
height.last.log	1.01	1.00	4.01	1.00	0.99	0.25	1.00
trail	1.06	1.03	1.13	1.03	0.94	0.89	0.97
damage	1.06	1.03	1.13	1.03	0.94	0.88	0.97
canopy openness	1.01	1.00	1.71	1.00	0.99	0.58	1.00
**(b) growth**							
height.last.log	1.00	1.00	Inf	1.00	1.00	0.00	1.00
trail	1.04	1.01	1.15	1.02	0.96	0.87	0.99
damage	1.04	1.01	1.15	1.02	0.96	0.87	0.99
canopy openness	1.02	1.00	1.32	1.01	0.98	0.76	1.00
**(c) recruitment**							
trail	1.03	1.00	1.34	1.02	0.97	0.74	1.00
damage	1.05	1.01	1.25	1.02	0.95	0.80	0.99
canopy openness	1.02	1.00	1.84	1.01	0.98	0.54	1.00

Table S4. Shows collinearity diagnostics for environmental factors included in models for (a) survival, (b) growth, and (c) recruitment. Variance inflation factors (VIF), tolerance values, and their 95% confidence intervals are shown for each term. All VIF values were <1.1 and all tolerance values were >0.9, which indicates low multicollinearity among predictors across all models.

Seedling performance models – logging & lianas

Logging

	(a) survival			(b) growth			(c) recruitment		
Predictors	*Risk Ratios*	*CI*	*p*	*Estimates*	*CI*	*p*	*Incidence Rate Ratios*	*CI*	*p*
(Intercept)	10.47	3.95 – 27.79	**<0.001**	0.65	0.60 – 0.70	**<0.001**	1.20	0.96 – 1.50	0.105
height last log	1.49	1.35 – 1.64	**<0.001**	-0.15	-0.16 – -0.15	**<0.001**			
[logged.2008]	1.01	0.69 – 1.48	0.963	0.09	0.07 – 0.12	**<0.001**	0.97	0.71 – 1.33	0.847
[logged.2018]	0.65	0.44 – 0.95	**0.025**	0.03	-0.00 – 0.06	0.077	0.79	0.57 – 1.09	0.151
[logged.2020]	0.47	0.32 – 0.68	**<0.001**	0.06	0.03 – 0.09	**<0.001**	0.54	0.38 – 0.75	**<0.001**
**Random Effects**									
σ^2	1.64			0.01			0.84		
τ_{00}	0.18 $_{plot.sub}$			0.00 $_{plot.sub}$			0.13 $_{plot}$		
	0.23 $_{plot}$			0.00 $_{plot}$					
	18.64 $_{spp3}$			0.01 $_{spp3}$					
ICC	0.92			0.51			0.14		
N	71 $_{spp3}$			68 $_{spp3}$			80 $_{plot}$		
	80 $_{plot}$			79 $_{plot}$					
	645 $_{plot.sub}$			553 $_{plot.sub}$					
Observations	2251			1595			720		
Marginal R^2 / Conditional R^2	0.010 / 0.921			0.352 / 0.682			0.059 / 0.189		

Table S5. Differences in (a) survival, (b) growth, and (c) recruitment in each logging treatment compared to unlogged forests. Linear mixed-effects models were used to test whether seedling performance was significantly different for subplots in each of the three logging treatments compared to unlogged forests. Random effects show the residual variance (σ^2), plot-level variance (τ_{00}), intraclass correlation coefficient (ICC), the number of plots (N), total observations, and the variance explained by fixed effects (Marginal R^2) and the full model (Conditional R^2).

Lianas

	(a) survival			(b) growth			(c) recruitment		
Predictors	*Risk Ratios*	*CI*	*p*	*Estimates*	*CI*	*p*	*Incidence Rate Ratios*	*CI*	*p*
(Intercept)	10.05	4.32 – 23.39	**<0.001**	0.66	0.62 – 0.71	**<0.001**	0.80	0.61 – 1.05	0.112
height last log	1.48	1.34 – 1.63	**<0.001**	-0.15	-0.16 – -0.15	**<0.001**			
plot type3 [logged.2008]	1.00	0.67 – 1.52	0.981	0.08	0.05 – 0.11	**<0.001**	0.88	0.60 – 1.31	0.542
plot type3 [logged.2018]	0.63	0.42 – 0.95	**0.028**	0.01	-0.02 – 0.05	0.411	0.55	0.36 – 0.83	**0.004**
plot type3 [logged.2020]	0.52	0.35 – 0.78	**0.002**	0.05	0.02 – 0.08	**0.004**	0.59	0.34 – 0.89	**0.011**
liana	1.11	0.81 – 1.52	0.507	-0.02	-0.05 – 0.00	0.066	0.45	0.33 – 1.62	**<0.001**
plot type3 [logged.2008] × liana	0.97	0.63 – 1.50	0.894	0.04	0.00 – 0.07	**0.029**	1.08	0.67 – 1.72	0.759
plot type3 [logged.2018] × liana	1.06	0.68 – 1.63	0.804	0.04	0.00 – 0.08	**0.038**	1.41	0.86 – 2.30	0.176
plot type3 [logged.2020] × liana	0.70	0.46 – 1.05	0.081	0.04	0.00 – 0.07	**0.047**	0.75	0.44 – 1.27	0.278
**Random Effects**									
σ^2	1.64			0.01			1.35		
τ_{00}	0.18 $_{plot.sub}$			0.00 $_{plot.sub}$			0.19 $_{plot}$		
	0.23 $_{plot}$			0.00 $_{plot}$					
	18.39 $_{spp3}$			0.01 $_{spp3}$					
ICC	0.92			0.50			0.12		
N	71 $_{spp3}$			68 $_{spp3}$			80 $_{plot}$		
	80 $_{plot}$			79 $_{plot}$					
	641 $_{plot.sub}$			548 $_{plot.sub}$					
Observations	2197			1562			1440		
Marginal R^2 / Conditional R^2	0.010 / 0.920			0.357 / 0.677			0.132 / 0.237		

Table S6. Differences in (a) survival, (b) growth, and (c) recruitment in lianas vs. trees overall and for lianas vs. trees in each logging treatment compared to unlogged forests. Linear mixed-effects models were used to test whether there were significant differences when comparing liana vs. seedling performance overall and in comparing whether liana vs. seedling performance was different in each logged forest treatment compared to the unlogged

forest treatments. Random effects show the residual variance (σ^2), plot-level variance (τ_{00}), intraclass correlation coefficient (ICC), the number of plots (N), total observations, and the variance explained by fixed effects (Marginal R^2) and the full model (Conditional R^2).
